# Characterization of the Worthen Sparrow (*Spizella wortheni*)’s Nest Building Materials in Northeastern Mexico

**DOI:** 10.3390/ani14081230

**Published:** 2024-04-19

**Authors:** Eliseo B. Suarez, Miguel Mellado, Marcos Luna, Eloy A. Lozano, Guadalupe Calderon, Yesenia Angel, Oscar Angel, Mayra L. Medina, José E. García

**Affiliations:** 1 Graduate Program in Sciences in Agriculture and Livestock Production, Autonomous Agrarian University Antonio Narro, Torreon 27054, Mexico; 2 Department of Animal Nutrition, Autonomous Agrarian University Antonio Narro, Saltillo 25315, Mexico; 3 Department of Renewable Natural Resources, Autonomous Agrarian University Antonio Narro, Saltillo 25315, Mexico; 4 Department of Veterinary Science, Autonomous Agrarian University Antonio Narro, Torreon 27054, Mexico; 5 Agricultural Technological Baccaluarte Center, No. 207, Viesca 27480, Mexico

**Keywords:** bird nesting ecology, desert, rangeland, plant–animal interaction, nest structure

## Abstract

**Simple Summary:**

The Worthen sparrow (WS) is an endemic species of the Mexican rangelands that is considered endangered. The objective of this study was to document the building materials for nest building of this obligate grassland bird species. After completing the breeding season from 2013–2016, 207 empty nests were collected to analyze the construction materials used in their assembly. The findings revealed that *Muhlembergia torrey* was a key component of the WS’s nest.

**Abstract:**

The study was conducted within a well-managed beef cattle operation in northeastern Mexico. Each nest was weighed and dissected to obtain the plant and animal material used to build the nests. The number of materials present per nest and relative frequency were determined. Twenty-one building materials were used. Over the years, *Muhlenbergia torreyi* represented 85.5% of the total biomass of the nests, and *Aristida longiseta*, *Bouteloua gracilis*, *Brickellia canescens*, *Purshia mexicana* and *Cirsium ehrenbergii* constituted 2.45, 2.80, 2.44, 1.34 and 1.11% of the total biomass, respectively. The above-mentioned grasses represented 95.62% of the total biomass. Material of animal origin was horse and cow hair, which represented 0.84 and 0.58% of the total biomass, respectively. It was concluded that, at the study site, *Muhlenbergia torreyi* was a key nest-building material for the Worthen sparrow nest.

## 1. Introduction

The Worthen sparrow (WS; *Spizella wortheni*) is a species endemic to the states of Nuevo León, Coahuila, Zacatecas, and San Luis Potosí in Mexico [1,2,3]. It does not carry out migratory movements and inhabits semi-desert areas in the grassland and scrub ecotone [1] associated with small shrubs [4]. Microphylous shrubland mixed with short grassland [5], rosetophilous shrublands dominated by the *Yucca* genus and creosote bush (*Larrea tridentata*), tarbush (*Fluorensia cernua*) as a dominant species at the shrub layer, and microphylous shrublands dominated by creosote bush and tarbush with grasses and annual plants [3,6] are habits wherein WSs prosper.

This grassland bird is found in the Official Gazette of the Federation and the Official Mexican Standard NOM-059 [7] and is a species at risk requiring environmental protection. The WS is considered globally to be in danger of extinction on the (IUCN) red list of threatened species due to its small population size and its limited range [3,8,9], as well as its low reproductive potential (14–25% reproductive success; [8,10]). In addition, it faces serious problems finding nesting sites due to overgrazing by livestock in northern Mexico and the defragmentation of its habitat by the opening of agricultural areas. Nothing is known about how the nesting success of the obligate grassland bird the WS is affected by habitat deterioration and the grazing of beef cattle, which reduce vegetation height and could affect the heterogeneity of the vegetation’s structure [11]. Additionally, it is not known if the WS uses animal hair to build their nest.

Very little is known about the materials used by most grassland-nesting birds. Basic information on plant and animal materials is needed to develop effective and practical management guidelines for prairie birds. The structural properties (e.g., rigidity), and insulative properties (animal’s hair) are basic variables that need to be characterized to understand the strategies of WS to avoid nest predation and brood parasitism and increase reproductive success.

Avian nests are multifaceted structures engineered to support incubation, shielding from adverse weather conditions, and chick rearing [12,13]. Additionally, materials used in nest building are crucial for shelter and reproduction [14,15]. The choices of materials in different parts of a nest probably reflect decisions made by the bird for the optimal development of its chicks [16]. Regarding bird nest construction, relatively few details are known for particular species [17], and few reports quantify the materials used in nests [18]. There is significantly more interest in studying the physical structure of the nest rather than in the behavior that produces it, which is a focus of the present study because of the logistical issues surrounding the building materials used by prairie birds [19].

The hypothesis was that the GW mainly uses grasses with not very lignified stems that are therefore malleable for building nests. This study aimed to describe in detail the materials utilized by the GW in constructing their nests in rangeland grazed by beef-producing cattle and with the presence of prairie dog colonies.

## 2. Materials and Methods

All animal procedures were approved by the Institutional Animal Care Committee and were handled following the guidelines outlined by the “Guide for Care and Use of Agricultural Animals in Research and Teaching” [20].

### 2.1. Study Area

**Figure 1:**
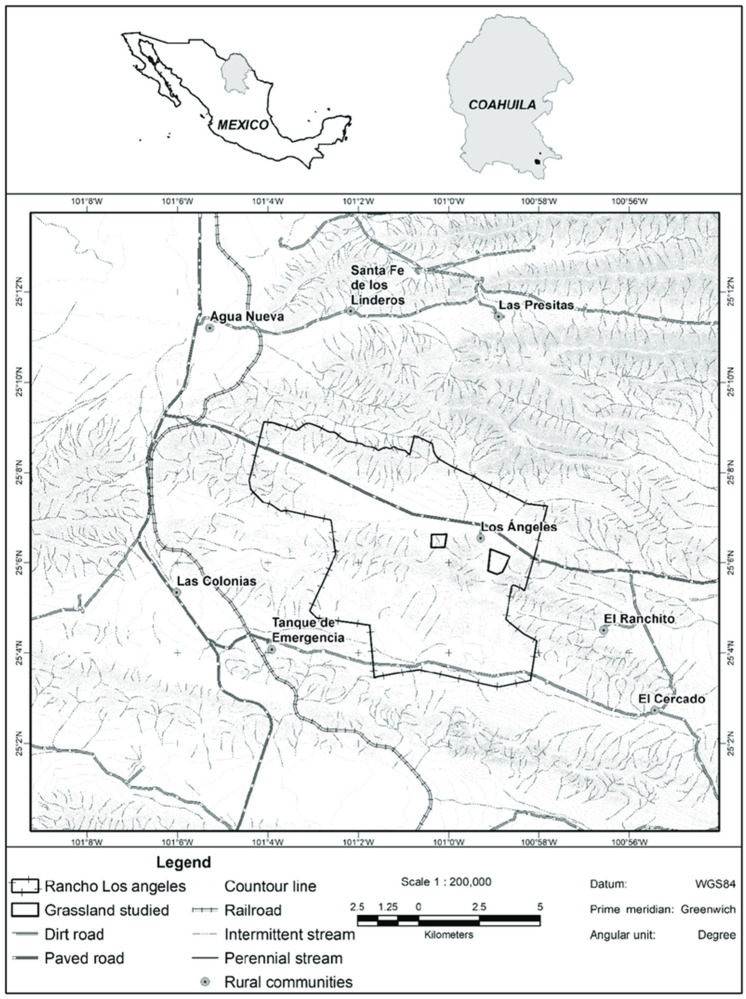
Geographic location of the study area: Los Angeles ranch, municipality of Saltillo, Mexico.

The study was carried out on the beef cattle ranch “Los Ángeles”, which is located 34 km from Saltillo, Coahuila, Mexico, between 25°04′12″ and 25°08′51″ north latitude and 100°58′07″ and 101°03′12″ west longitude (Figure 1), with an average altitude of 2150 m.

The study site covers an approximate area of 6700 ha. According to [21], the climate is semi-arid, with cool winters, an average annual temperature of 15.5 °C, and an average annual precipitation of 450 mm. The surrounding and dominant grassland in the native semi-desert study area is the buffalo grass (*Bouteloua dactyloides*) (Nutt.) Columbus, with dominant species such as *Muhlenbergia phleoides* (Kunth) Columbus, *M. arenicola* Buckley, *Aristida arizonica* Vasey, *Erioneurum avenaceum* (Kunth) Tateoka, *Bouteloua gracilis* (Willd. ex Kunth) Lag. ex Griffiths. Primary forb species include *Tiquilia canescens* (DC.) Richardson, *Croton dioicus* Cav., *Zinnia acerosa* (DC.) Gray, *Solanum elaeagnifolium* Cav., *Sphaeralcea angustifolia* (Cav.) D. Don, and *Dychoriste linearis* (Torr. & A. Gray) Kuntze. Shrubs such as *Flourensia cernua* DC., *Opuntia engelmanii* Salm–Dyck, and *Cylindropuntia imbricata* (Haw.) F.M. grow in isolated patches. *Buddleja scordioides* Kunth is another important shrub in this landscape.

### 2.2. WS Nesting Characterization

Given that the breeding season of the WS is from May to July, WS nesting was characterized during the spring and summer of 2013 to 2016. A total of 207 empty WS nests were collected in a desert microphyllous scrub. To locate the nests, groups of 3 to 4 people walked parallel to a distance of no more than 2 m to facilitate the discovery of nests of this bird species. Field observers located the nests by hearing the WSs’ singing; they minimized the conspicuousness of observers and nests to predators during searching and monitoring [22].

Walks were carried out throughout the day in places known for the reproduction of the WS, especially in areas with the presence of colonies of prairie dogs (*Cynomys mexicanus*) in microphyllous scrub vegetation. WS is closely associated with habitats where Mexican prairie dog (*Cynomys mexicanus*) colonies occur, and therefore, WS are inclined to build their nests in shrub-grassland areas inhabited by these colony-forming rodent species [4].

Once a nest was located, it was georeferenced using Normal Mercator Projection coordinates, and we recorded on which plant or substrate the nest was found. Then, visible tape was placed on a bush no less than five meters away near the nest to facilitate its immediate location after the reproduction season. The nests were collected upon their abandonment.

### 2.3. Identification of Grassland Plants and Other Species

The collected nests were stored in sealed paper bags and dried at 60 °C in a laboratory dry plant oven for three days. The entire nest was weighed on an electronic scale. Each nest was carefully dissected to separate natural materials, after which each material type was weighed. Plant or animal materials were then qualitatively identified (composition and possible source material) to quantify nest materials. Each plant or animal material that made up the nest was weighed. The outer walls of the nest were removed first until the lower limit of the outer part of the cup was reached. The cup was then removed to leave the base of the external nest by separating elements with tweezers. Samples were weighed with a precision balance (Acculab Pocket Pro C/50 ^©^ Merck KGaA, Darmstadt, Germany) to the nearest 0.002 g to determine the dry mass of each component of the nest.

Before deconstruction, nests were visually examined to identify the body, base, and nest bed. The main easily identifiable regions were the outer part, consisting of rigid plant material pieces, and the central parts, base, and bed.

Plant species were identified by observing certain distinguishing morphological characteristics of the stem, leaf, bud, flower, and fruit. For those plants that could not be identified in the laboratory, samples of these species were placed in a plant press, labeled, and sent to the university herbarium for identification.

### 2.4. Statistical Analyses

The nest’s weight between years was analyzed using the GLM procedure of SAS (SAS Inst. Inc., Cary, NC, USA; version 9.4) with individual nests as the experimental units. Inter-site variation was not tested because most nests were in the same habitat. Descriptive statistics were used to present the materials used for nest building.

## 3. Results

It was observed that both males and female participated in the building of their nest. WS nests found per year were 29 in 2013, 55 in 2014, 71 in 2015, and 52 in 2016, with a total of 207 nests.

**Figure 2:**
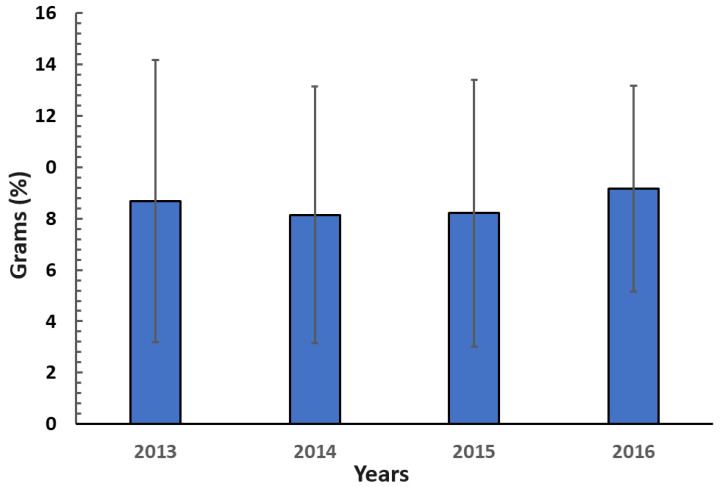
The total weight of Worthen sparrow nests recorded over four years in desert rangeland in northeastern Mexico. Values are means ± SD.

Figure 2 shows the average weight of the GW nests in the different sampling years. There was no significant difference (*p* > 0.05) in nest weight between years. High values of standard deviations of nest weight indicated wide variation in nest weight across all years. The materials used in the construction of the nests are presented in Table 1. Nineteen plants (mostly grasses) and cow’s and horse’s hair were used as nesting materials by the WS. However, *Muhlenbergia torreyi* made up the bulk of the WS’s nest, and only traces of many grasses were used as building materials.

When the frequency of the occurrence of the different construction materials of the 207 nests analyzed was considered, *Muhlenbergia torreyi* was present in all the nests (Table 2). *Bouteloua gracilis* was present in half of the nests, while *Aristida longiseta* and *Brickellia canescens* occurred in a third of the nests. The presence of *Bouteloua curtipendula* and *Muhlenbergia repens* as materials for manufacturing WS nests was negligible.

**Figure 3:**
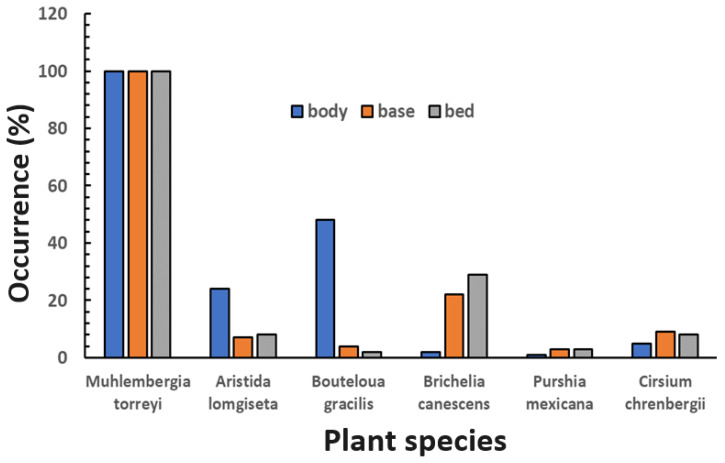
Occurrence of construction materials in different parts of Worthen sparrow nests in rangeland in northeastern Mexico.

When considering the nest’s body, base, and bed, *Muhlenbergia torreyi* was present in all these parts of the nest (Figure 2). *Bouteloua gracilis* was important in the body of the nest, but this grass was practically not used for other parts of the nest. *Brickellia canescens* was an essential plant in nest bed construction (Figure 3).

## 4. Discussion

It has been reported that grassland birds have greater sensitivity to vegetation structure than to the particular composition of plant species [23]. The WS follows this pattern since it is not dependent on a specific group of shrubs [2,24] and possibly prefers the vegetation structure of the habitat where the study was carried out. The above-mentioned fact can be inferred from the large number of nests found, which far exceeds the values previously reported for the WS [2,25] in habitats of northern Mexico. In the study area, the types of vegetation that the WS prefers were present, such as tarbush (*Flourensia cernua*) [26] and mariola (*Parthenium incanum*) [27]. It has been observed that WS increased with increasing yuca density and mean shrub height [28].

The average height of shrubs is believed to be related to the nesting substrate. Previous studies have found that WSs build their nests in shrubs with a height of 25 to 95 cm [2,27,29]. Therefore, the positive response in habitat occupancy to shrub height recorded in the present study may respond to the species’ dependence on the shrub stratum for nesting and possibly foraging. The WS, like other birds from similar habitats, perhaps uses bushes of approximately 1 m to place their nests for protection from predators or nest parasites [30,31].

*Muhlenbergia torreyi* was identified as a fundamental material for the construction of the WS nests. This grass is not the most abundant in this rangeland [32], yet the WS selected it over many other abundant grasses in this ecosystem. According to the ‘availability hypothesis’, birds simply select the building materials most widely available, and this is backed by the fact that nest composition varies according to the availability of nesting materials in the surroundings [33]. The data from the present study do not support this hypothesis since WS mainly selected *Muhlenbergia torreyi* for the different areas of the nest. Both bovine and equine hair were negligible components of the nest, which implies that these materials were not chosen for their insulative properties, as has been the case in other prairie birds [34]. WSs seem to depend on dry grasses to line the cup, a material that significantly correlates with the insulation properties of the nest [35].

Nest construction by the WS exhibited high repeatability between years, which aligns with [36]. Nests, built by most birds, are essential for reproductive success, so some species spend considerable time and energy searching for particular materials to construct their nests [37]. This appears to be the case with the WSs, which were highly selective when choosing construction materials. Material choices within different parts of a nest reflect decisions made by the birds and appear to have a structural role [38]. WSs selected at least 21 materials, mainly grasses. 

Structural analysis of WS nests showed that the base of the outer nests was composed of significantly thicker, stronger, and more rigid materials than the inner nest wall and nest base. Despite differences in the rigidity of different parts of the nest, *Muhlebergia torreyi* was present in all sections of the nest. This fact suggests that WS selected stems of *M. torrey* of various diameters for different nest parts. Securing warmth and safety are most important for small birds and their young, which explains why birds of small size generally build more elaborate nests than larger birds [39] and why their nests have greater thermal insulation [40]. It is possible that the WS selected the short grass *M. torrey*, which has slender stems suitable for waving the nest and appressed stems that would facilitate the collection of this material. The presence of *Brickellia canescens*, particularly on the base of the nest, was probably sought as an insulating material, given the woolly nature of this plant’s leaves and upper stems. These data showed that grasses provided good material for cup nest building for these small birds, providing adequate protection for their young and resisting stresses.

Future research directions can be oriented to the amount of foliage or complexity of habitat surrounding the nest of WSs and how the height of the nest may influence vulnerability to brood parasitism or nest predation by affecting predator movement. Additionally, it would be interesting to determine vegetation characteristics in the nest patches, the concealment of the nest in patches, and the thickness of support branches. These results provide biologists with critical information on what vegetal and animal materials are used by WS, indicating that nest construction behavior is not plastic in this species due to the abundance of building materials in this landscape; they also show how few grass species solve the problem of building a WS nest that will accommodate incubation and allow the successful hatching of eggs.

## 5. Conclusions

It was concluded that the Worthen sparrow, an obligate grassland bird, appeared to be more successful in our study area than in most other areas of northern Mexico wherein their nesting has been evaluated. *Muhlembergia torrey* was a key component of the Worthen sparrow’s nest. These data contribute to a database that sets a baseline for the nest composition of the Worthen sparrow, which may prove helpful in future studies. Further observations are necessary in order to better understand nest construction in the context of evolutionary trade-offs, thermoregulation, and predation.

## Figures and Tables

**Table 1:** Plant species and animal materials contributing to the biomass of the Worthen sparrows’ nests in the rangeland of northeastern Mexico.

Scientific Name	Biomass (g)	Biomass (%)
*Muhlenbergia torreyi*	7.27	85.48
*Aristida longiseta*	0.25	2.95
*Bouteloua gracilis*	0.24	2.80
*Brickellia canescens*	0.21	2.44
*Purshia mexicana*	0.11	1.34
*Cirsium ehrenbergii*	0.09	1.11
*Horsehair*	0.07	0.84
Unknown (rootlets)	0.06	0.69
*Stipa nassella*	0.05	0.63
*Cow hair*	0.05	0.58
*Solidago glutinosa*	0.03	0.29
*Descurania pinnata*	0.02	0.26
*Elymus elymoides*	0.01	0.11
*Solidago missourensis*	0.01	0.11
*Lepidium virginicum*	0.01	0.11
*Eragrostis mexicana*	0.01	0.09
*Leresquelia*	0.01	0.07
*Scleropogon brevifolius*	traces	0.05
*Muhlenbergia repens*	traces	0.02
*Panicum halii*	traces	0.02
*Bouteloua curtipendula*		0.01
Total	-	100

**Table 2:** Frequency of Worthen sparrow nesting materials in a rangeland in northeastern Mexico.

Scientific Name	Number of Nests	Frequency, %
*Muhlenbergia torreyi*	207	100
*Bouteloua gracilis*	100	48.30
*Aristida longiseta*	81	3913
*Brickellia canescens*	71	34.29
*Purshia mexicana*	40	19.32
*Horsehair*	36	17.39
*Solidago glutinoso*	27	13.04
Cow hair	20	9.66
*Stipa nassella*	15	7.24
*Cirsium ehrenbergii*	14	6.76
Unknown (rootlets)	13	6.28
*Elymus elymoides*	12	5.79
*Descurania pinnata*	8	3.86
*Solidago missourensis*	7	3.38
*Eragrostis mexicana*	6	2.89
*Lepidium virginicum*	5	2.41
*Bouteloua curtipendula*	4	1.93
*Muhlenbergia repens*	2	0.96
*Panicum halii*	2	0.96
*Lesquerella* spp.	1	0.48
*Scleropogon brevifolius*	1	0.48

## Data Availability

Data supporting this study are available from J.E. García upon reasonable request.
